# *Undariopsis peterseniana* Promotes Hair Growth by the Activation of Wnt/β-Catenin and ERK Pathways

**DOI:** 10.3390/md15050130

**Published:** 2017-05-05

**Authors:** Jung-Il Kang, Min-Kyoung Kim, Ji-Hyeok Lee, You-Jin Jeon, Eun-Kyoung Hwang, Young-Sang Koh, Jin-Won Hyun, Soon-Young Kwon, Eun-Sook Yoo, Hee-Kyoung Kang

**Affiliations:** 1 Department of Medicine, School of Medicine, Jeju National University, 102 Jejudaehakno, Jeju 63243, Korea; jikang0024@jejunu.ac.kr (J.-I.K.); loveis6776@hanmail.net (M.-K.K.); yskoh7@jejunu.ac.kr (Y.-S.K.); jinwonh@jejunu.ac.kr (J.-W.H.); yonse2004@hanmail.net (S.-Y.K.); eunsyoo@jejunu.ac.kr (E.-S.Y.); 2 Department of Marine Life Science, Jeju National University, 102 Jejudaehakno, Jeju 63243, Korea; lee198186@hanmail.net (J.-H.L.); youjinj@jejunu.ac.kr (Y.-J.J.); 3 Aqua Green Technology Co. Ltd., 209 Jeju Bio-Industry Center, 102 Jejudaehakno, Jeju 63243, Korea; 4 Seaweed Research Center, National Institute of Fisheries Science, 130 Tongilro, Mokpo 58746, Korea; ekhwang@hotmail.com; 5 Jeju Research Center for Natural Medicine, Jeju National University, 102 Jejudaehakno, Jeju 63243, Korea

**Keywords:** *Undariopsis peterseniana*, hair growth, K_ATP_ channel, 5α-reductase, dermal papilla cell, Wnt/β-catenin, apo-9′-fucoxanthinone

## Abstract

In this study, we investigated the effect and mechanism of *Undariopsis peterseniana*, an edible brown alga, on hair growth. The treatment of vibrissa follicles with *U. peterseniana* extract ex vivo for 21 days significantly increased the hair-fiber lengths. The *U. peterseniana* extract also significantly accelerated anagen initiation in vivo. Moreover, we found that *U. peterseniana* extract was able to open the K_ATP_ channel, which may contribute to increased hair growth. The *U. peterseniana* extract decreased 5α-reductase activity and markedly increased the proliferation of dermal papilla cells, a central regulator of the hair cycle. The *U. peterseniana* extract increased the levels of cell cycle proteins, such as Cyclin D1, phospho(ser780)-pRB, Cyclin E, phospho-CDK2, and CDK2. The *U. peterseniana* extract also increased the phosphorylation of ERK and the levels of Wnt/β-catenin signaling proteins such as glycogen synthase kinase-3β (GSK-3β) and β-catenin. These results suggested that the *U. peterseniana* extract had the potential to influence hair growth by dermal papilla cells proliferation through the activation of the Wnt/β-catenin and ERK pathways. We isolated a principal of the *U. peterseniana* extract, which was subsequently identified as apo-9′-fucoxanthinone, a trichogenic compound. The results suggested that *U. peterseniana* extract may have a pivotal role in the treatment of alopecia.

## 1. Introduction

Hair loss (alopecia) is a distressing disorder and has several contributory factors, including the imbalance of hormones, stress, poor nutrition, and chemotherapy [1,2,3]. Two drugs, minoxidil (Rogain^®^) and finasteride (Propecia^®^), have been approved by the Food and Drug Administration to alleviate hair loss [4,5]. Minoxidil, a K_ATP_ channel opener, was originally used to treat hypertension [4,6], but has also been shown to cure alopecia [6]. Finasteride has been widely used to cure prostatic hypertrophy and androgenetic alopecia [5,6]. However, when it is used for the treatment of patients with alopecia, weaknesses arise, including transient action and infertility problems [6,7]. Therefore, there is a high demand for other substances that may be used for the treatment of alopecia.

The hair follicle is a small organ that undergoes structural changes depending on the hair cycle, such as in the growth (anagen), regression (catagen), and resting (telogen) phases [8]. Throughout the hair cycle, the growth of hair follicles (HF) is regulated by the interaction between adjacent hair follicle cells, including dermal papilla and stem cells (SC) [9]. In particular, dermal papilla cells, mesenchymal derived fibroblasts, operate as a central regulator in the hair cycle [10]. At the end of telogen phase, the signals from the dermal papilla are transmitted to SC and/or hair germ cells to initiate hair growth [10]. Studies on hair growth have shown that the proliferation of dermal papilla cells was accompanied by increase in the duration of the anagen phase [11]. Several signaling proteins, such as Wnt/β-catenin and extracellular signal regulated kinases (ERK), were upregulated in dermal papilla cells after minoxidil treatment and led to the proliferation of dermal papilla cells [11,12]; this was accompanied by the alteration of cell cycle proteins, including cyclin E, cyclin dependent kinases 2 (CDK2), and p27^Kip1^ [13]. The Wnt/β-catenin pathway is crucial in both hair growth and follicle development [14,15]. A study on the role of β-catenin, a component of the Wnt/β-catenin pathway, observed hair loss in mice lacking β-catenin [15], which suggested that alteration of the Wnt/β-catenin pathway might induce hair growth or alopecia. The ERK pathway has been shown to affect cellular functions including cell proliferation and apoptosis [16,17]. Several activators of hair-growth, such as the vascular endothelial growth factor, placental growth factor, and adenosine, have displayed ERK-mediated hair growth effects [18,19].

*Undariopsis peterseniana*, an edible brown alga with a rich source of nutrients such as amino acids and minerals, is found in abundance on the coast of Jeju Island, Korea. Several studies have focused on the cultivation of *U. peterseniana* [20,21]. The antioxidant activity of *U. peterseniana* has also been demonstrated by using the DPPH radical scavenging assay [22]. However, the effects of *U. peterseniana* on hair growth, and the underlying mechanisms of action, have not been investigated. This study investigated the in vitro, ex vivo, and in vivo effects and mechanism of *U. peterseniana* extract on hair growth.

## 2. Results

### 2.1. Ex Vivo Effect of U. peterseniana Extract on the Hair-Fiber Length of Rat Vibrissa Follicles

**Figure 1:**
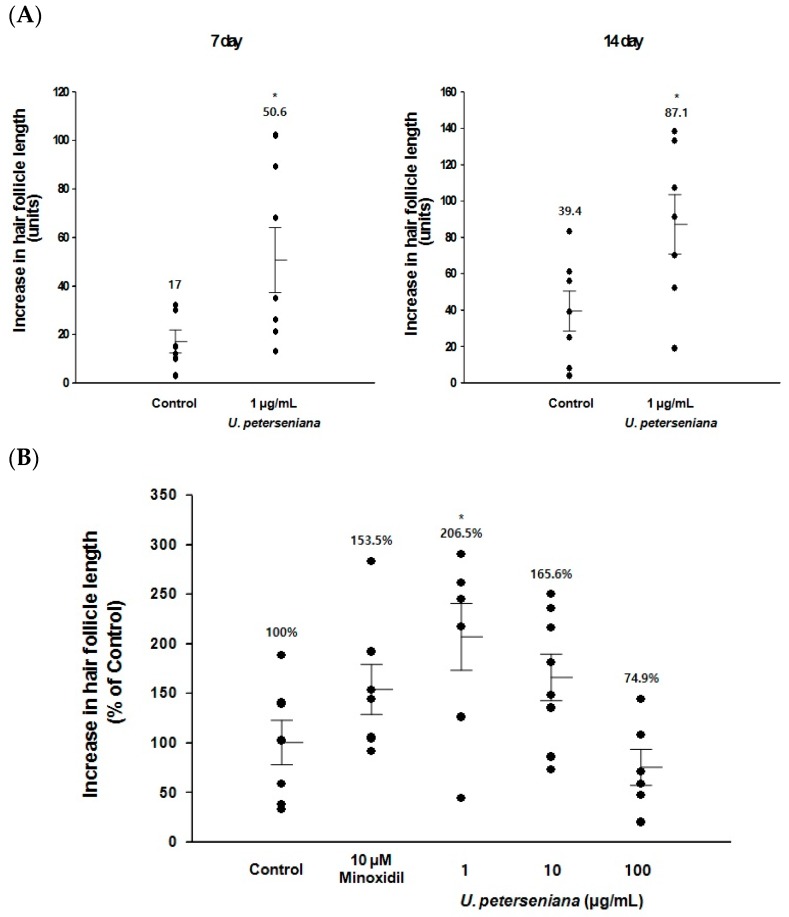
*Undariopsis peterseniana* extract increases hair growth in ex vivo organ culture. Rat vibrissa follicles were cultured with the indicated concentrations of *U. peterseniana* extract or minoxidil for 21 days. (**A**) The time course change of hair-fiber length was measured by DP controller software. Each dot indicates an independent follicle length. Horizontal lines showed mean ± SE. * p < 0.05 compared with the control; (**B**) The change in length of vibrissa follicles at 21 days was compared to the mean length of control follicles. The mean length of control follicles was set at 100% and each dot indicates an independent follicle length (%). Horizontal lines showed the mean ± SE. * p < 0.05 compared with the control.

To test the effect of *U. peterseniana* extract on hair growth, we evaluated the change in hair-fiber length using organ cultures of rat vibrissa follicles [23]. Isolated rat vibrissa follicles were treated with *U. peterseniana* extract (1, 10, and 100 μg/mL) for 21 days and the hair-fiber length was measured on days 0, 7, 14, and 21 after isolation. The hair-fiber lengths of the vibrissa follicles treated with *U. peterseniana* extract (1 μg/mL) significantly increased after 7 days; the increase continued for 21 days (Figure 1A,B). On day 21, *U. peterseniana* extract (1 and 10 µg/mL) had increased the hair-fiber length by 206.5% and 165.6%, respectively, compared with vehicle-treated control (Figure 1B). In particular, 1 µg/mL *U. peterseniana* extract significantly increased the hair-fiber length compared with the positive control of 10 μM minoxidil, whereas 100 μg/mL *U. peterseniana* extract did not affect the hair-fiber length (Figure 1B).

### 2.2. Effect of U. peterseniana Extract on the Telegen to Anagen Progression in C57BL/6 Mouse

**Figure 2:**
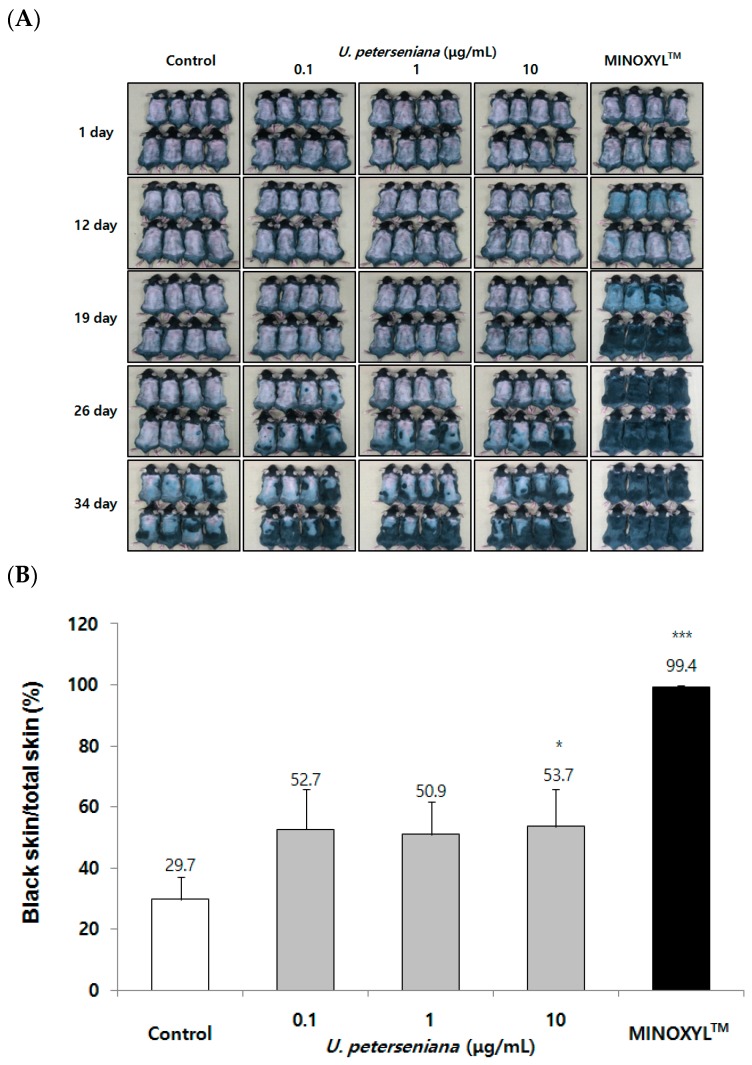
*U. peterseniana* extract accelerates the progression of anagen phase in vivo. *U. peterseniana* extract (0.1, 1, and 10 μg/mL) was topically treated to C57BL/6 mice dorsal skin for 34 days. (**A**) The change in skin color was observed after photographing at the indicated times after depilation; (**B**) Quantification of anagen induction by dotmatrix planimetry. On day 34, the area of interest in back skin was marked in transparency film. Acceleration to the anagen phase was expressed as a percentage of the area of the black skin compare to the total skin area. Data are presented as the mean ± SE (*n* = 8). * p < 0.05, *** p < 0.001 compared with the control.

In order to evaluate the in vivo effect on hair growth, C57BL/6 mice whose hair cycle was consistent with changes in skin color were used for in vivo experiments [8]. More specifically, it is known that skin color changes from pink to black when the hair cycle progress from telogen to anagen in previous studies [8]. *U. peterseniana* extract (0.1, 1, and 10 μg/mL) was topically applied to the skin of C57BL/6 mice from 1 day after shaving and the treatment was applied daily for 34 days. On the day 34, *U. peterseniana* extract induced gray/black skin, but the vehicle-treated control was visibly less pigmented (Figure 2A). On day 34 after treatment with *U. peterseniana* extract, the quantification of anagen progression was analyzed using dotmatrix planimetry [24]. The 10 µg/mL *U. peterseniana* extract-treated mice showed larger gray/black skin area than vehicle-treated mice (Figure 2B). From day 12, the skin color of MINOXYL™ (5% minoxidil)-treated mice changed to gray, and after 26 days, the hair was fully grown (Figure 2A). These results indicated that *U. peterseniana* extract significantly induced transition of the hair cycle from the telogen phase to the anagen phase in vivo.

### 2.3. Effect of U. peterseniana Extract on the 5α-Reductase Activity 

**Figure 3:**
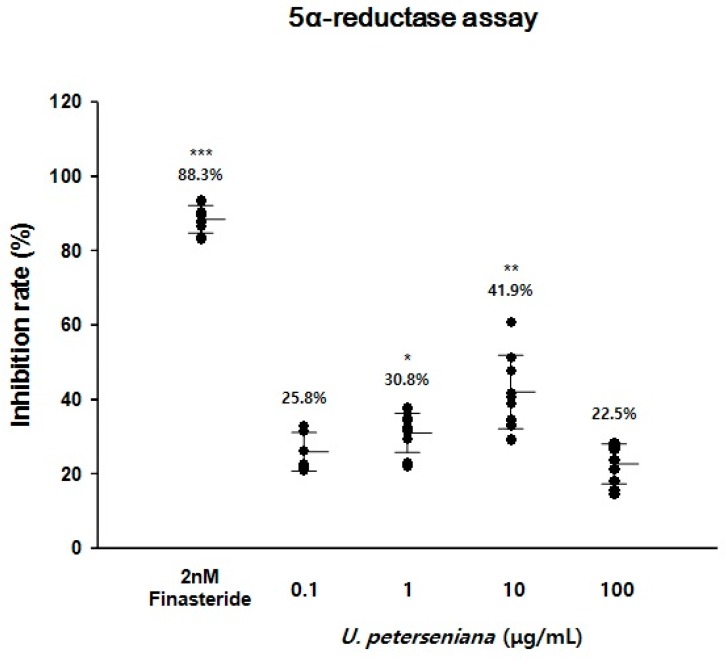
*U. peterseniana* extract inhibits 5α-reductase activity. 5α-Reductase activity was measured by liquid scintillation counting using [1,2,6,7-^3^H] testosterone. The reaction mixtures were incubated with the indicated concentrations of *U. peterseniana* extract or finasteride in the presence of crude extract of rat prostate. The conversion rate of testosterone (T) to dihydrotestosterone (DHT) was calculated by the equation: [DHT/(T + DHT)] × 100. The inhibition rate of 5α-reductase activity (%) was estimated as a percentage of the reduction in conversion rate compared with the control. The inhibition rate of the control group was considered 0% (not shown). * *p* < 0.05, **p < 0.01, *** *p* < 0.001 compared with the control. Horizontal lines show the mean ± SD. Finasteride was used as reference material to evaluate the inhibition of 5α-reductase activity.

5α-Reductase has been reported to be highly expressed in HF of androgenetic alopecia patients [25]. Clinical trials have revealed that treatment with finasteride, a type II 5α-Reductase inhibitor, led to the prevention of hair loss [5]. To evaluate whether *U. peterseniana* extract could inhibit 5α-reductase activity, we used a crude 5α-reductase from rat prostate. The addition of *U. peterseniana* extract (0.1, 1, 10, and 100 µg/mL) to the reaction mixture inhibited 5α-reductase activity by 25.8%, 30.8% (*p* < 0.05), 41.9% (*p* < 0.01), and 22.5%, respectively (Figure 3). Finatseride (2 nM) also significantly inhibited the 5α-reductase activity by 88.3% (*p* < 0.001) (Figure 3). These results suggested that *U. peterseniana* extract may have the potential to treat androgenetic alopecia; however, the 5α-reductase activity of *U. peterseniana* extract was lower than that of finasteride.

### 2.4. Effect of U. peterseniana Extract on the Proliferation of NIH3T3 Fibroblasts via K_ATP_ Channel Opening

Minoxidil, a K_ATP_ channel opener, has a mitogenic effect on NIH3T3 fibroblasts [26]. The 3-[4,5-dimethylthiazol-2-yl]-2,5-diphenyltetrazolium bromide (MTT) assay revealed that minoxidil-induced proliferation of NIH3T3 fibroblasts was inhibited by K_ATP_ channel blockers [26].

**Figure 4:**
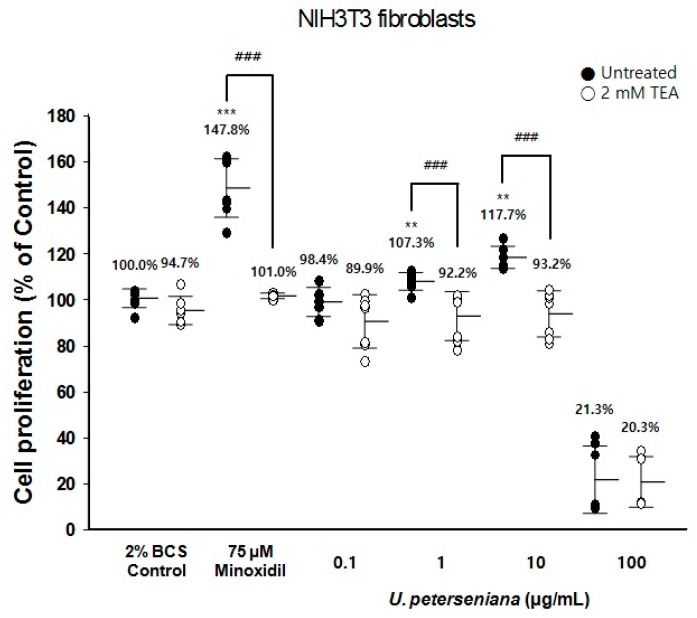
Effect of *U. peterseniana* extract on the proliferation of NIH3T3 fibroblast via the opening of K_ATP_ channel. The proliferation of NIH3T3 fibroblasts by the opening of K_ATP_ channel was measured by MTT assay and tetraethylammonium (TEA) was used as a K_ATP_ channel blocker. NIH3T3 fibroblasts were pretreated with or without 2 mM TEA, and treated with various concentrations of *U. peterseniana* extract for 4 days. The proliferation of NIH3T3 fibroblasts was evaluated compared with the 2% BCS control. Each dot indicates an independent experiment and horizontal lines show the mean ± SD. Indicated concentration of minoxidil and TEA were used as reference materials to evaluate cell proliferation by K_ATP_ channel. ** p < 0.01, *** p < 0.001 compared with the control; ^###^ *p* < 0.001 compared with the TEA-treated group.

To address whether *U. peterseniana* extract acted as an opener of the K_ATP_ channel, we investigated the proliferation of NIH3T3 fibroblasts using tetraethylammonium (TEA), a K_ATP_ channel blocker, by an MTT assay. *U. peterseniana* extract (1 and 10 µg/mL) significantly increased the proliferation of NIH3T3 fibroblasts compared with the vehicle-treated control (107.3% and 117.7%, respectively) (Figure 4). TEA (2 mM) completely inhibited the increase in the proliferation of NIH3T3 fibroblasts caused by *U. peterseniana* extract. Minoxidil increased the proliferation of NIH3T3 fibroblasts by 147.8% at a concentration of 75 µM; this increase was also completely inhibited by 2 mM TEA (Figure 4). These data indicated that the opening of the K_ATP_ channel led to an increase in the proliferation of NIH3T3 fibroblasts. The results indicated that *U. peterseniana* extract could act as a K_ATP_ channel opener, which could be a contributory factor in the effect on hair growth.

### 2.5. Effects of U. peterseniana Extract on the Proliferation of Dermal Papilla Cells

**Figure 5:**
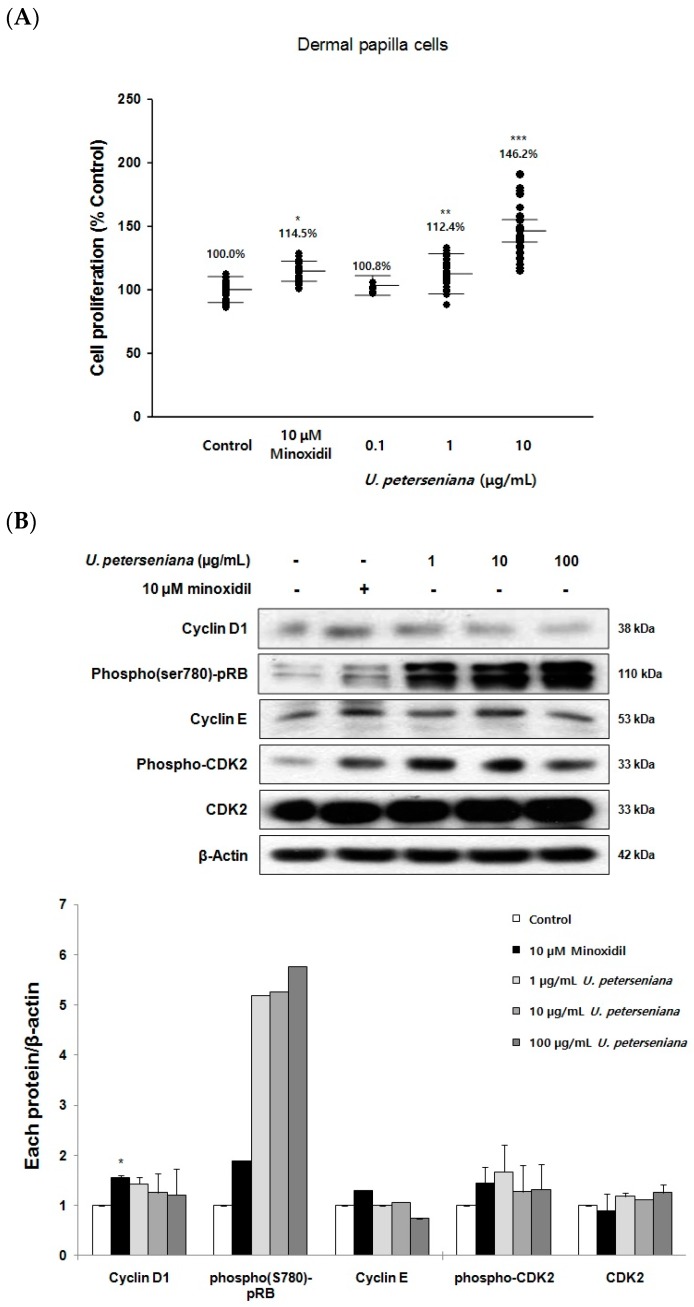

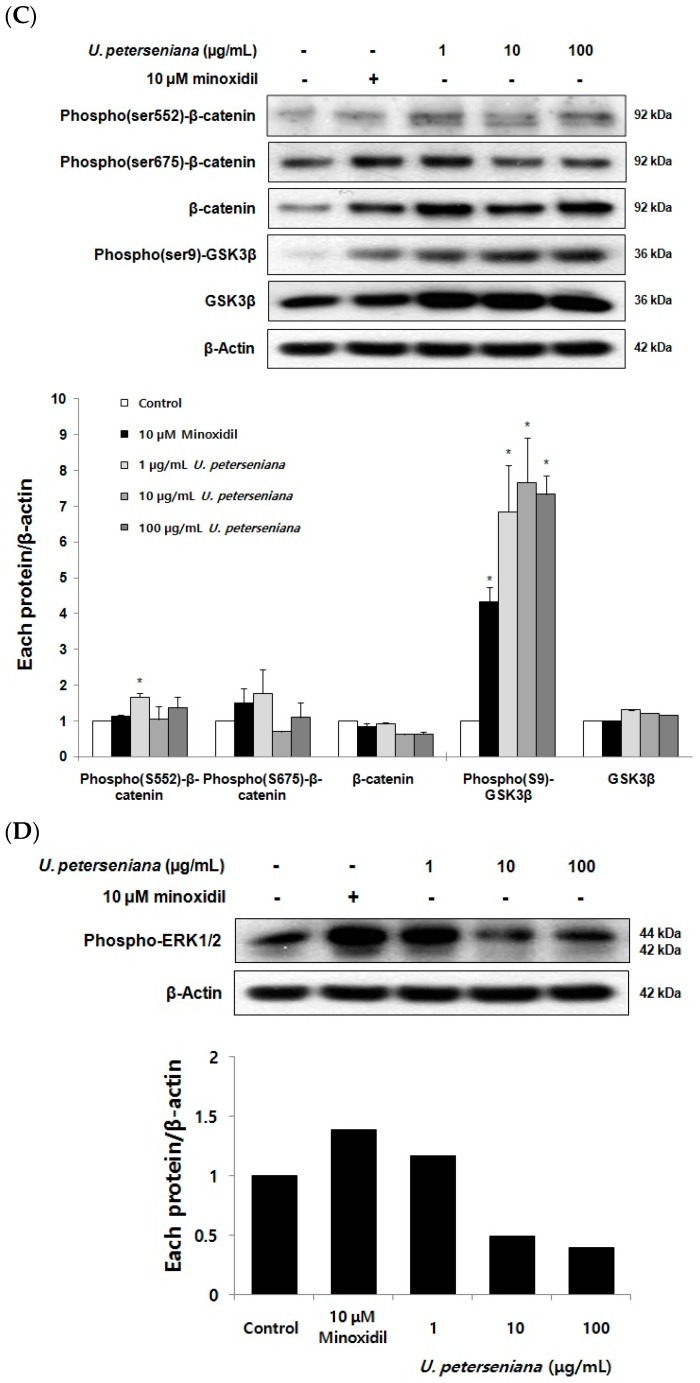
*U. peterseniana* extract increases the proliferation of dermal papilla cells as well as the levels of different proteins (cell cycle proteins, Wnt/β-catenin, and phospho-ERK) in dermal papilla cells. (**A**) The dermal papilla cells were stimulated with *U. peterseniana* extract and minoxidil, indicated concentrations for 96 h. The MTT assay was used to assess the proliferation of dermal papilla cells. Each dot indicates an independent experiment and horizontal lines showed the mean ± SD. * *p* < 0.05, **p < 0.01, *** *p* < 0.001 compared with the control; (**B**–**D**) The dermal papilla cells (1.0 × 10^6^ cells) were stimulated *U. peterseniana* extract and minoxidil, indicated concentrations for 24 h. The whole cell lysate from dermal papilla cells were subjected to immunoblotting with indicated antibodies. * *p* < 0.05 compared with the control.

To test the effect of *U. peterseniana* extract on hair follicular cells, the proliferation of dermal papilla cells was measured by the MTT assay after incubation with *U. peterseniana* extract (0.1, 1, and 10 µg/mL) for 96 h. *U. peterseniana* extract (1 and 10 µg/mL) significantly increased the proliferation of dermal papilla cells by 112.4% (*p* < 0.01) and 146.2% (*p* < 0.001), respectively (Figure 5A). The positive control, minoxidil (10 µM) significantly increased the proliferation of dermal papilla cells by 114.5% (*p* < 0.05) compared with the control (Figure 5A). These results suggested that *U. peterseniana* extract could exert a promotional effect on hair-growth via the proliferation of dermal papilla cells.

### 2.6. Effects of U. peterseniana Extract on the Levels of Cell Cycle Proteins, Wnt/β-Catenin Signaling, and Erk1/2 Signaling in Dermal Papilla Cells

The progression of the cell cycle is required for cell proliferation and is driven by cell cycle-related proteins (cyclin/CDKs complexes and CDK inhibitors) [27]. To investigate whether *U. peterseniana* extract increased the proliferation of dermal papilla cells by the regulation of cell cycle-related proteins, we examined changes in the levels of Cyclin D1, phospho(ser780)-pRB, Cyclin E, phospho-CDK2, and CDK2 after treatment with *U. peterseniana* extract (1, 10, and 100 µg/mL) for 24 h in dermal papilla cells. *U. peterseniana* extract increased the levels of Cyclin D1, phospho(ser780)-pRB, Cyclin E, phospho-CDK2, and CDK2 (Figure 5B). 

The Wnt/β-catenin pathway is crucial for the proliferation of dermal papilla cells and hair growth [28]. Therefore, we addressed whether *U. peterseniana* extract could activate the Wnt/β-catenin pathway. We examined the changes in the levels of phosphorylation of β-catenin at ser552, 675, phosphorylation of GSK-3β at ser9, and GSK-3β following the treatment of *U. peterseniana* extract for 24 h. *U. peterseniana* extract increased the levels of phosphorylation of β-catenin at ser552, 675, phosphorylation of GSK-3β at ser9, and GSK-3β in dermal papilla cells (Figure 5C). The treatment with minoxidil (10 µM) resulted in an increase in the levels of phosphorylation of β-catenin at ser552, 675, β-catenin, and phosphorylation of GSK-3β at ser9 compared with the vehicle-treated control (Figure 5C). The data showed that minoxidil and *U. peterseniana* extract increased the level of β-catenin in the nucleus through the regulation of GSK-3β [11]. The results indicated that *U. peterseniana* extract could increase the proliferation of dermal papilla cells via the activation of the Wnt/β-catenin pathway.

The ERK pathway is also important in cell proliferation [16]; the inhibition of ERK has been shown to result in a decrease in cell proliferation [29]. We therefore examined the changes in the level of phospho-ERK1/2 after the treatment of *U. peterseniana* extract. The increase of phospho-Erk1/2 level was observed in cells treated with *U. peterseniana* extract (Figure 5D), which indicated that the activation of ERK pathway by *U. peterseniana* extract contributed to the proliferation of dermal papilla cells.

## 3. Discussion

In the present study, we demonstrated that *U. peterseniana* extract promoted the growth of hair by facilitating both the elongation of hair-fiber length and initiation of anagen. Moreover, we observed that in addition to the inhibition of 5α-reductase activity and K_ATP_ channel opening, *U. peterseniana* extract could induce the proliferation of dermal papilla cells via the activation of Wnt/β-catenin and ERK pathways. 

**Figure 6:**
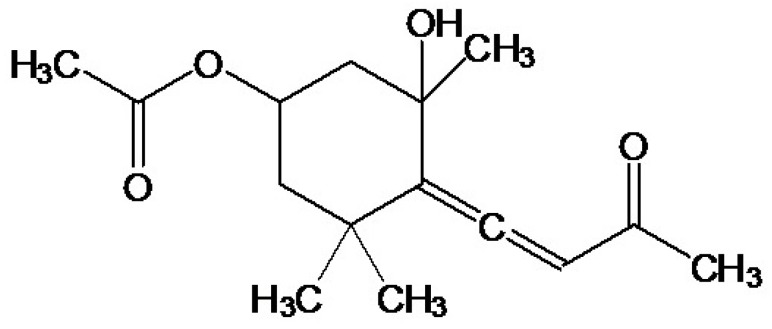
Structure of apo-9′-fucoxanthinone isolated from *U. peterseniana*.

A previous study determined that 5α-reductase converted T to DHT, a potent form of T, which induced the progression of alopecia [5]. Inhibition of 5α-reductase activity in alopecia patients decreased the level of DHT in the scalp, which led to a decrease in the progression of alopecia [30]. Finasteride, the type II 5α-reductase inhibitor, was shown to cure androgenetic alopecia by a reduction of DHT level [5]. In this study, *U. peterseniana* extract displayed an inhibitory effect on 5α-reductase activity, which suggested that *U. peterseniana* extract has the potential to prevent androgenetic alopecia by the inhibition of 5α-reductase activity. Apo-9′-fucoxanthinone, a principal compound of brown algae including *U. peterseniana*, has also been shown to inhibit 5α-reductase activity (Figure 6) [31,32].

K_ATP_ channels are expressed in HF and the channel opening has been linked to cell proliferation and hair growth [26,33]. In this study, the proliferation of NIH3T3 fibroblasts in response to *U. peterseniana* extract was completely mitigated by TEA, a K_ATP_ channel blocker. The role of K_ATP_ channel opening in response to minoxidil was supported by the finding that treatment with TEA blocked the minoxidil-induced proliferation of NIH3T3 fibroblasts [26]. These results indicated that *U. peterseniana* extract had the potential to increase cell proliferation by opening the K_ATP_ channels. Apo-9′-fucoxanthinone was found to be a principal of both the *U. peterseniana* extract and *Sargassum muticum* extract [31,32]. Nevertheless, on the proliferation of NIH3T3 fibroblast via the opening of K_ATP_ channel, *U. peterseniana* extract (1 and 10 µg/mL) significantly increased the proliferation of NIH3T3 fibroblasts, whereas apo-9′-fucoxanthinone could not change that (data not shown). To explain the discrepancy of effects between *U. peterseniana* extract and apo-9′-fucoxanthinone, we need to find the other components from *U. peterseniana* extract.

We investigated the effect of *U. peterseniana* extract on the increase of hair-fiber length as an ex vivo experiment. The culture of HF has many advantages: at least two distinct aspects such as the measure of hair-fiber length and maintenance of normal architecture [23,34]. A previous study showed that minoxidil increased not only the length of hair-fiber in the organ culture of mouse HF, but also thymidine incorporation [34]. Likewise, our findings revealed that the hair-fiber lengths of follicles stimulated with *U. peterseniana* extract (1 µg/mL) were significantly longer than those of follicles treated with minoxidil, which has been used in hair research models both ex vivo and in vivo as a positive control. It has previously been suggested that the progression of the hair cycle was confirmed by a change in skin color from pink to gray/black [8,24]. Treatment with *U. peterseniana* extract also boosted anagen initiation in C57BL/6 mice. The results indicated that *U. peterseniana* extract had the potential to promote hair growth by the partial regulation of distinct effects that resulted from the K_ATP_ channel opening and the inhibition of 5α-reductase activity. Except for the results of the length of hair-fiber in culture of HF, the other results showed that the *U. peterseniana* extract (10 μg/mL) exhibited significant effects. According to our previous research [32], a possible explanation for this was as follows: in the experiment on the culture of HF, the culture medium containing *U. peterseniana* extract was changed every 3 days, and this practice was continued for 21 days. Therefore, the effective concentration was lower than expected, because *U. peterseniana* extract and its metabolites were concentrated in HF.

The size and number of dermal papilla cells located at the base of HF are known to be related to hair cycle and hair growth [35]. During the hair cycle, dermal papilla cells are known to interact with hair follicle SC and hair germ cells and to mediate the transition from telogen to anagen in the hair cycle by the regulation of the signaling pathway, including fibroblast growth factors and Wnt/β-catenin proteins, which led to an early onset of anagen phase [10].

Cell cycle control is important for cell proliferation [27]. The role of cell cycle promoting proteins including phospho-pRB and cyclins is to induce the cell cycle progression from G0/1 to S phase; Cell cycle inhibitory proteins (CDK inhibitors) prevent this process [27]. In the present study, *U. peterseniana* extract upregulated the level of cyclin D1, phospho-pRB, cyclin E, phospho-CDK2, and CDK2, followed by the proliferation of dermal papilla cells. We previously demonstrated that *Ishige sinicola*, a brown alga, or minoxidil, could increase the levels of cyclin E and CDK2, but decreased the level of the p27^kip1^, a CDK inhibitor in dermal papilla cells [36]. This was further supported by the observation that glibenclamide, a K_ATP_ channel blocker, inhibited cell proliferation, which was accompanied by an increase in cell cycle arrest and the p27^kip1^ level [37]. These results suggested that *U. peterseniana* extract increased the proliferation of dermal papilla cells through the progression of the cell cycle by the alteration of the level of cell cycle proteins. On the other hand, on the proliferation of dermal papilla cells, the efficacy of *U. peterseniana* extract (146.2%) was higher than that of apo-9′-fucoxanthinone (121.7%), an active component of the *U. peterseniana* extract. In further study, we need to isolate other components in order to address the discrepancy of effects between *U. peterseniana* extract and apo-9′-fucoxanthinone.

The Wnt/β-catenin pathway is one of the master regulator of cell growth in several cell types, including hair follicle SC and pancreatic beta cells [28,38], and is inhibited by GSK-3β [39,40]. Previous findings suggested that the inactivation of GSK-3β by LiCl, an inhibitor of GSK-3β, led to a decrease in the ubiquitination of β-catenin, which resulted in the phosphorylation and translocation of β-catenin into the nucleus [40]. The increased level of β-catenin in the nucleus activates target genes such as cyclin D1, thereby allowing cell proliferation [41]. In several studies, natural products have been reported to increase hair growth by Wnt/β-catenin activation [42,43]. Our study revealed that *U. peterseniana* extract enhanced the levels of phosphorylation of GSK-3β at ser9 and phosphorylation of β-catenin at ser552, 675 in dermal papilla cells. Apo-9'-fucoxanthinone, an active compound of *U. peterseniana*, was reported to increase the levels of phosphorylation of GSK-3β at ser9 and phosphorylation of β-catenin at ser552 [32]. The Wnt/β-catenin activation by *U. peterseniana* extract and apo-9′-fucoxanthinone is involved in the proliferation of dermal papilla cells. However, our study did not address what type of kinase regulated the phosphorylation of Wnt/β-catenin proteins, although the activation of Wnt/β-catenin proteins are known to be regulated by protein kinase A and protein kinase B [40,44,45]. Further studies using specific inhibitors are necessary to reveal the detailed mechanism.

The activation of the ERK pathway is important for the proliferation of various cell types including dermal papilla cells [16,46]. In this study, we showed that *U. peterseniana* extract increased the phosphorylation of ERK in dermal papilla cells. Endothelin, a mitogen of airway smooth muscle, increased cell proliferation, which was inhibited by PD98059, an ERK inhibitor [29]. Our results indicated that *U. peterseniana* extract has the potential to increase the proliferation of dermal papilla cells through the regulation of ERK phosphorylation. In addition, a direct association between the upregulation of cyclin D1 and the phosphorylation of ERK has been reported [47]. As mentioned above, our findings showed that *U. peterseniana* extract increased the levels of cyclin D1, β-catenin, an upstream regulator of cyclin D1, and the phosphorylation of ERK in dermal papilla cells, which suggested that *U. peterseniana* extract increased the proliferation of dermal papilla cells through the regulation of the levels of cell cycle proteins, Wnt/β-catenin proteins, and the phosphorylation of ERK. 

In the study, we identified the hair growth-promoting effects of *U. peterseniana* via the proliferation of dermal papilla cells, opening of K_ATP_ channels, and the inhibition of 5α-reductase activity. Furthermore, *U. peterseniana* extract could induce the proliferation of dermal papilla cells via the increase of cell cycle proteins and the activation of Wnt/β-catenin and ERK pathways. We also identified apo-9′-fucoxanthinone as an active component of *U. peterseniana* extract. Collectively, these results suggested that *U. peterseniana* extract may be used for the development of a new therapeutic substance for the treatment of alopecia.

## 4. Materials and Methods

### 4.1. Reagents

Earle’s balanced salt solution (EBSS), dimethyl sulfoxide (DMSO), dithiothreitol (DTT), hydrocortisone, insulin, minoxidil, phosphate-buffered saline (PBS), phenylmethylsulfonylfluoride (PMSF), tetraethylammonium (TEA) and MTT were purchased from Sigma-Aldrich (St. Louis, MO, USA). Fetal bovine serum (FBS), bovine calf serum (BCS), Williams medium E, l-glutamine and penicillin/streptomycin solution (Pen Strep) were purchased from Gibco (Gibco Life Technologies, Grand Island, NY, USA). Dulbecco’s modification of Eagle’s medium was purchase from Hyclone (Logan, UT, USA). Aprotinin and leupeptin were purchased from Calbiochem (San Diego, CA, USA).

### 4.2. Preparation of Undariopsis peterseniana Extract

*Undariopsis peterseniana* was collected along the coast of Jeju Island, Korea, between April and July 2010 and identified by Dr Eun-Kyoung Hwang. A voucher specimen (SRI-P00017) was deposited at the herbarium of the Seaweed Research Center, National Fisheries Research and Development Institute. The sample was washed with distilled water, and stored at −20 °C. The frozen sample was finely homogenized with a grinder. The 15 kg of *U. peterseniana* was extracted with the three sample volumes of 70% ethanol and then concentrated using a vacuum evaporator. The resulting residue (600 g) was stored at −20 °C for use in subsequent experiments. *U. peterseniana* extract was dissolved in DMSO at a concentration of 50 mg/mL and stored at −20 °C until use. From the *U. peterseniana* extract, we isolated apo-9′-fucoxanthinone by a centrifugal partition chromatography (CPC) system and preparative thin layer chromatography. The purified apo-9′-fucoxanthinone was identified by a comparison of the ^1^H- and ^13^C-NMR data with those reported in the literature [31].

### 4.3. Animals

Three-week-old male Wistar rats, six-week-old female C57BL/6 mice, and seven-week-old male Sprague-Dawley (SD) rats were purchased from Orient Bio (Seongnam, Gyeonggi, Korea) and provided with a standard laboratory diet and water *ad libitum*. All animals were cared for by using protocols (20100031) approved by the Institutional Animal Care and Use Committee (IACUC) of Jeju National University.

### 4.4. Isolation and Culture of Rat Vibrissa Follicles

The isolation of rat vibrissa follicles was performed using the method of Philpott and Kealey [23]. Male Wistar rats (23 day old) were sacrificed under carbon dioxide (CO_2_), and the mystacial pads were removed and placed in P/E buffer (1:1 mixture of PBS and EBSS that contained 1% Pen Strep). Anagen vibrissa follicles were carefully isolated from mystacial pads using sterile dissecting forceps and blade while being observed under a stereomicroscope (Olympus, Tokyo, Japan). The isolated follicles were cultured in Williams medium E (Gibco Life Technologies, Grand Island, NY, USA) supplemented with 2 mM l-glutamine (Gibco Inc., Grand Island, NY, USA), 50 nM hydrocortisone, 10 µg/mL insulin and 1% Pen Strep at 37 °C in an 5% CO_2_/95% air. Minoxidil was dissolved in 0.12 mM HCl at a concentration of 5 mM and used as the positive control [33]. The culture medium containing the *U. peterseniana* extract (1, 10, and 100 µg/mL) or 10 µM minoxidil was changed every 3 days. The lengths of the HF were measured using DP controller software ver. 1.1.1.65 (Olympus, Tokyo, Japan). 

### 4.5. Hair Growth Activity In Vivo

The anagen phase of hair cycle was induced by removing hair on the back skin of C57BL/6 mice, as described previously [8]. For anagen induction, the back skin of female C57BL/6 mice (P49) was shaved by animal clipper. *U. peterseniana* (0.2 mL) extract treatment began at P50, with daily administration for 34 days. Mice were photographed at 1, 12, 19, 26, and 34 days while observing changes in skin color on the back skin of the mice. Quantitative results were obtained using dotmatrix planimetry [24].

### 4.6. Assay for Prostatic 5α-Reductase Activity

To prepare rat prostates, 8-week-old male SD rats were sacrificed with CO_2_. The prostates were separated from surrounding capsules, washed twice with PBS, and stored at −80 °C. The weight of frozen prostate was measured. The five tissue volume of homogenase buffer (20 mM potassium phosphate buffer (pH 6.6), 0.32 M sucrose, 25 μg/mL leupeptin, 25 μg/mL aprotinin, 1 mM DTT, and 0.2 mM PMSF) was added to prostate and homogenized with a taco™ Prep Bead Beater (GeneReach Corp., Taichung, Taiwan). The homogenates were centrifuged at 100,000× *g* for 60 min. The obtained pellets were suspended in homogenase buffer and stored at −80 °C until use. 5α-Reductase activities were analyzed by the measurement of the radioactivity using a liquid scintillation counter (LSC, Packard Bioscience, Meriden, CT, USA) as follows [48]. The 5α-reductase reaction was initiated by the adding 250 μg of prostatic enzyme fraction and *U. peterseniana* extract (0.1, 1, 10, and 100 μg/mL) in reaction buffer (buffer A). The buffer A contained 40 mM potassium phosphate buffer (pH 6.6), 2 mM NADPH, and 120 nCi (1,2,6,7-^3^H) T and protease inhibitor cocktail. Three independent reactions were examined and finasteride was used as a positive control to confirm the inhibition of type II 5α-reductase activity. The enzyme reaction was carried out at 37 °C for 1 h, and ethyl acetate (EtOAc) was added to stop the reaction. After the reaction, the sample was centrifuged at 1000× *g* for 5 min, and the supernatant was transferred to a new tube. After drying on a heating plate, 50 μL of EtOAc containing T (500 μg/mL) and DHT (500 μg/mL) was added to dissolve the residues. The sample was applied to a silica gel 60 F254 TLC plate and developed with a developing solvent (1:1 mixture of EtOAc:cyclohexane). After drying at 25 °C, the TLC spot of T was observed under UV light (254 nm). The TLC spot of DHT was visualized by soaking in 10% H_2_SO_4_ solution and heating with an alcohol lamp. The spot portion of T and DHT were clipped off and then dissolved in the ULTIMA GOLD™ Cocktail (5 mL). The activity of 5α-reductase measured by LSC was expressed as a ratio calculated by the equation: [DHT/(T + DHT)] × 100.

### 4.7. Cell Culture

Rat vibrissa immortalized dermal papilla cell line [49] was donated by the Skin Research Institute, Amore Pacific Corporation R&D Center, South Korea. The mouse embryonic NIH3T3 fibroblasts were purchased from ATCC (Rockville, MD, USA). Dermal papilla cells were cultured in DMEM (Hyclone Inc., Logan, UT, USA) supplemented with 10% heat-inactivated fetal bovine serum (FBS) (Gibco-BRL Inc., Long Island, NY, USA) and 1% Pen Strep at 37 °C in a 5% CO_2_/95% air. NIH3T3 fibroblasts were cultured in ATCC-formulated DMEM supplemented with 10% heat-inactivated bovine calf serum (BCS and 1% Pen Strep at 37 °C in a 5% CO_2_/95% air.

### 4.8. Proliferation Assay of NIH3T3 Fibroblasts

The MTT assay was used to determine the cell proliferation as described previously [50]. NIH3T3 fibroblasts were seeded at 2000 cells per well in 96-well plate in DMEM supplemented with 2% BCS. The cell were incubated for 24 h to allow attachment, *U. peterseniana* extract (0.1, 1, 10, and 100 μg/mL) and minoxidil (75 μM) were treated with cells for 96 h. To investigate whether the cell proliferation was regulated through the K_ATP_ channel opening, the cells were pretreated with or without 2 mM TEA for 2 h and then treated with *U. peterseniana* extract (0.1, 1, 10, and 100 μg/mL) and 75 μM of minoxidil. After 96 h, MTT (0.1 mg) was added to each well, and the cells were cultured at 37 °C in a 5% CO_2_/95% air for 4 h. In sequence, the media was carefully discarded and 200 µL of DMSO was added to the well. The optical density was measured at 540 nm on a Versamax microplate reader (Molecular Devices, Sunnyvale, CA, USA). All experiments were repeated at least three times and the results were expressed as the percentage change compared to the average absorbance of the vehicle-treated controls. 

### 4.9. Proliferation Assay of Dermal Papilla Cells

The MTT assay was used to determine the cell proliferation as described previously [50]. The MTT assays were performed as follows: dermal papilla cells were seeded at 2000 cells per well in 96-well plates in DMEM supplemented with 1% FBS. The cells were incubated for 24 h to allow attachment, and were stimulated with *U. peterseniana* extract (0.1, 1, and 10 μg/mL) or 10 μM minoxidil. After 96 h, MTT (0.1 mg) was added to each well, and the cells were cultured at 37 °C in a 5% CO_2_/95% air for 4 h. Next, the media was carefully discarded and 200 µL of DMSO was added to the well. The optical density was measured at 540 nm on a Versamax microplate reader (Molecular Devices, Sunnyvale, CA, USA). All experiments were repeated at least three times and the results were expressed as the percentage change compared to the average absorbance of the vehicle-treated controls.

### 4.10. Western Blot Analysis

The dermal papilla cells were seeded at 1.0 × 10^6^ cells per 100-mm dish in DMEM supplemented with 1% FBS and the cells were cultured at 37 °C in a 5% CO_2_/95% air. After 24 h, the cells were treated with *U. peterseniana* extract (0.1, 1, and 10 μg/mL) and 10 μM minoxidil. The cells were lysed and the protein concentration was determined using a Bio-rad protein assay dye, based on the Bradford method [51]. The proteins (20~30 μg/well) were separated on 10~12% SDS-PAGE gels and then transferred onto PVDF membranes. After blocking with 5% nonfat dried milk in TBS containing 0.1% Tween-20 (TBST), each membrane was incubated with specific primary antibodies (Table 1) overnight at 4 °C. The membranes were washed six times with TBST and incubated with HRP-conjugated secondary antibody (1:5000) at 25 °C for 1 h. The antigen/antibody complexes visualized using West-zol™ Plus and the Image J software were used to quantify the intensity of the band [52].

### 4.11. Statistical Analysis

Data were expressed as the mean ± standard deviation (SD) or standard error (SE) of at least triplicate experiments. The statistical differences between experimental and control groups were estimated using Student’s *t*-test. The statistical analysis was performed using SigmaStat Software ver. 3.5 (San Jose, CA, USA). A *p*-value < 0.05 was considered statistically significant.

## Figures and Tables

**Table 1:** List of antibodies used for immunoblotting.

Antibodies	Supplier	Species	dilution
phospho-(ser552)-β-catenin	Cell Signaling	Rabbit	1:1000
phospho-(ser675)-β-catenin	Cell Signaling	Rabbit	1:1000
β-catenin	Santa Cruz	Rabbit	1:2000
phospho(ser9)-GSK-3β	Cell Signaling	Rabbit	1:1000
GSK-3β	Cell Signaling	Rabbit	1:1000
phospho-Erk1/2	Cell Signaling	Rabbit	1:1000
Cyclin D1	BD Biosciences	Mouse	1:1000
phospho(ser780)-pRB	Cell Signaling	Rabbit	1:1000
Cyclin E	Santa Cruz	Rabbit	1:1000
phospho-CDK2	Cell Signaling	Rabbit	1:1000
CDK2	Santa Cruz	Rabbit	1:1000
β-actin	Sigma-Aldrich	Mouse	1:5000
